# Supplementary material for: The effect of mutations derived from mouse-adapted H3N2 seasonal influenza A virus to pathogenicity and host adaptation
Source: PLoS One. 2020 Jan 9;15(1):e0227516. doi: 10.1371/journal.pone.0227516 (PMC6952113; doi:10.1371/journal.pone.0227516)
Supplement: S1 Table — Balb/c mice were inoculated with SW293, B_P8_C2, or B_P9_C2, and the live and dead mice were counted daily for nine days. The 50% mouse lethal dose (MLD50) was calculated using Reed-Muench method. (PDF) [file pone.0227516.s001.pdf]

Supplemental Data

S1 Table. MLD_{50} of Balb/c-passaged clones in Balb/c mice

Virus	Titer (PFU/mL)	No. of dead mice	No. of survival mice	Log MLD_{50} (PFU/mL)
SW293	2.79×10^7	0	4	> 7.45
	2.79×10^6	0	5	
	2.79×10^5	0	5	
	2.79×10^4	0	5	
	2.79×10^3	0	5	
	2.79×10^2	0	5	
B_P8_C2	7.88×10^7	5	0	4.40
	7.88×10^6	5	0	
	7.88×10^5	5	0	
	7.88×10^4	5	0	
	7.88×10^3	0	5	
	7.88×10^2	0	5	
B_P9_C2	4.78×10^7	5	0	4.30
	4.78×10^6	5	0	
	4.78×10^5	4	0	
	4.78×10^4	4	1	
	4.78×10^3	0	5	
	4.78×10^2	0	5	

Balb/c mice were inoculated with SW293, B_P8_C2, or B_P9_C2, and the live and dead mice were counted daily for nine days. The 50% mouse lethal dose (MLD_{50}) was calculated using Reed-Muench method.
