# Supplementary material for: The effect of mutations derived from mouse-adapted H3N2 seasonal influenza A virus to pathogenicity and host adaptation
Source: PLoS One. 2020 Jan 9;15(1):e0227516. doi: 10.1371/journal.pone.0227516 (PMC6952113; doi:10.1371/journal.pone.0227516)
Supplement: Attachment — Submitted filename: Rebuttal.doc [file pone.0227516.s002.doc]

**Responses to reviewer comments**

**Responses to reviewer comments on PONE-D-19-21391**

**[Reviewer #1]**

**Major point 1:** A figure showing increase of viral titer in mice as the number of passage increases is recommended.

**Response:** Following the reviewer’s suggestion, we added Tables 1-4, showing mice mortality after serial infection with the seasonal H3N2 influenza (A/Switzerland/9715293/2013, SW293). In addition, the process of generating the mouse-adapted H3N2 virus [maSW293B8 (MA_SW)] has been included in the revised manuscript.

*“To increase the virulence of seasonal H3N2 influenza in mice, SW293 was serially inoculated in a series of Balb/c or DBA/1J mice, and then the mouse mortality rates were monitored for nine days (Tables 1 and 2). Despite performing ten lung-cell-lung passages in Balb/c mice, animals were still not susceptible to the SW293 strain (Table 1). However, after three lung-cell-lung passages in DBA/1J mice, the virus was able to kill all mice in the group (Table 2). Thereafter, lung-to-lung passages were performed using DBA/1J mice until passage 13, and four clones each were acquired from passages 9, 11, 12, and 13 by plaque purification (D_P9_C1–4, D_P11_C1–4, D_P12_C1–4, D_P13_C1–4).”* (p. 10, lines 185–192)

*“For Balb/c mice, adaptation was initiated by infecting DBA/1J_P12_C1, which was followed by ten lung-to-lung passages (Table 3). After five passages, all inoculated mice died; therefore, four clones each from passage 6, 8, and 9 were obtained (B_P6_C1–4, B_P8_C1–4, and B_P9_C1–4). Five mice per group were infected intranasally with 10^6^ PFU of Balb/c-passaged clones and mortality rates were monitored for nine days (Table 4). At least four out of the five clone-infected mice died except for B_P8_C1. In particular, all mice infected with B_P8_C2 died, and 80% of them died before reaching the euthanasia baseline (Table 4). In addition, the MLD_50_ values of the Balb/c-passaged clones were about 1000-fold lower than that of the SW293 parental virus (S1 Table). These results demonstrate that the serial passage of SW293 parental virus results in markedly increased mortality and virulence in mice.”* (p. 11–12, lines 202–211)

*“We generated a mouse-adapted H3N2 virus [maSW293B8 (MA_SW)] derived from A/Switzerland/9715293/2013 by serial lung-cell-lung passages in Balb/c and DBA/1J mice, which resulted in higher mortality rates in mice (Table 4 and S1 Table).”* (p. 21, lines 372–374)

**Major point 2:** Was each mutation from each individual colony or all from a single colony? How many colonies were picked? In how many colonies was each mutation found? This information may be added to Table 1. If all from a single colony, different combinations of mutations in addition to single mutations should have been studied. Description of the process of identifying the mutations is recommended.

**Response:** Four Balb/c-passaged clones each were isolated from passages 6, 8, and 9 by plaque purification (B_P6_C1–4, B_P8_C1–4, and B_P9_C1–4). Since B_P8_C2 showed high pathogenicity and mortality in mice (Table 4 and S1 Table), reassortant H3N2 viruses were generated based on the mutations of this virus. Therefore, amino acid substitutions used in this study to find determinants of pathogenicity and host adaptation of influenza A virus were derived from the B_P8_C2 clone. This experimental method encompassing description of the process of identifying the mutations is described in subsections ‘Plaque purification’ and ‘DNA sequencing’ of the M&M section. All related data were mentioned in the results section and were added to Tables 4 and 5.

*“To isolate single-phenotype viruses, plaque purification was performed with lung isolates of each passage using MDCK cells, as described previously [28]. Briefly, supernatants of lung homogenates were serially diluted 10-fold in PBS from 10^−2^ to 10^−6^. Confluent MDCK cells were prepared in 6-well plates and infected with 200 μL of the diluted samples. After 1 h, the cells were washed with PBS and overlaid with 2 mL 0.8% agarose–medium mixture containing 1 μg/mL L-1-tosylamide-2-phenylmethyl chloromethyl ketone (TPCK)-treated trypsin (Thermo Fisher Scientific, Waltham, MA, USA). Three to four days later, single-plaque colonies were selected from each plate, resuspended in medium, and propagated in MDCK cells. After 48–72 h of incubation, viruses were harvested, and the viral titer (PFU/mL) of each strain was calculated.”* (p. 6–7, lines 106–114)

*“Viral RNA from each sample was isolated using the QIAamp® Viral RNA Mini kit (Qiagen, Hilden, Germany) following the manufacturer’s instructions. Reverse transcription of viral RNA was performed using the SuperScript III Reverse Transcriptase (Thermo Fisher Scientific). Subsequently, all gene segments were amplified using SuperTaq™ Plus Polymerase (Thermo Fisher Scientific). The PCR products were purified using the QIAquick® Gel Extraction kit (Qiagen). Sequencing was performed using a 3730 DNA analyzer (Applied Biosystems, Foster City, CA, USA), and the sequencing results for all viruses were analyzed and aligned using the CLC Main Workbench software.”* (p. 7, lines 117–124)

*“For Balb/c mice, adaptation was initiated by infecting DBA/1J_P12_C1, which was followed by ten lung-to-lung passages (Table 3). After five passages, all inoculated mice died; therefore, four clones each from passage 6, 8, and 9 were obtained (B_P6_C1–4, B_P8_C1–4, and B_P9_C1–4).”* (p. 11, lines 202–204)

*“To identify the mutations responsible for the increased ability to cause fatal infection, we initially sequenced the genomes of two Balb/c– and three DBA/1J–passaged clones (Table 5).”* (p. 13, lines 221–222)

We agree with the reviewers' comment that combinations of mutations derived from different genes are necessary to understand the interactions and synergy between the mouse-adapted genes and is mentioned in the discussion section (p. 24, lines 446–469). In this study, however, we focused on generating mouse-adapted strains of seasonal H3N2 influenza and examined the contribution of each gene derived from the mouse-adapted H3N2 seasonal virus to increasing its pathogenicity and host adaptation. Seasonal H3N2 influenza poorly replicates in mouse lung, making it difficult to generate mouse-adapted strains with high virulence in mice. Thus, the studies on mouse-adapted H3N2 seasonal strains and their mutations have been relatively rare compared to other subtypes. Although different combinations of mutations have been left as a follow-up study, we believe that our study is meaningful as a study inquiring the pathogenic determinants of the H3N2 virus.

**Major point 3:** Growth curves of the subject viruses (mutants and MA_SW) in MDCK cells and A549 cells are recommended. This is to see if the mutations were simple growth adaptations or host adaptations. The authors mention viral growths in MDCK cells in many places of the manuscript without actually showing the data.

**Response:** Following the reviewer’s suggestion, the growth curve of mutants, RG_SW, and MA_SW were confirmed in MDCK cells and are presented as new Fig 4. This experiment is described in the M&M and the results sections.

*“To analyze the time-dependent growth profiles of reassortant H3N2 viruses, MDCK cells were infected at a multiplicity of infection (MOI) of 0.001 and were incubated at 37°C. The supernatants were harvested at 3, 6, 12, 24, 48, and 72 h. The infectious viral titers were determined by TCID_50_ in MDCK cells.”* (p. 9, lines 164–167)

*“Cell growth properties of reassortant H3N2 viruses were examined in MDCK cells at 3, 6, 12, 24, 48, and 72 h post-infection (hpi) (Fig 4). All reassortant viruses and MA_SW showed higher peak titer and faster growth rate than those of RG_SW. The HA mutant presented the highest peak titer at 24 hpi and the fastest growth rate among the reassortant H3N2 viruses. The initial growth rate of the NP mutant was moderate; however, it recorded the second-highest peak titer following the HA mutant. The viral titer of the PB2 mutant was similar to that of the NP mutant until 24 hpi, after which the titer was maintained.* *These results indicated that mouse-adapted mutations contribute to the increased replication ability in mammalian cells.”* (p. 19–20, lines 325–332)

**Major point 4:** Since the input titers of the mutant viruses and MA_SW are different, the input titers of the mutants being 100-fold higher than MA_SW, it is difficult to determine the connection between each mutation and the MA characteristics. According to Fig 1 and 2, the mutants didn’t show MA characteristics at the titer where MA_SW was infected. Fig 3 and 4 cannot be interpreted properly for the same reason. To the reviewer, it appears that none of the mutations individually have notable MA characteristics. The authors didn’t make any effort to figure out which combination of the mutations recapitulates the MA_SW phenotype.

**Response:** Following the comment of the reviewer, the sentence “We found that the MA_SW titer was lower than that of other viruses (3.16×10^4^ TCID_50_/mL), suggesting that infection with >10^4^ TCID_50_ was limited.” was modified in the results section.

*“In the case of MA_SW, higher doses were not used as 10^3^ TCID_50_ showed sufficient lethality in mice.”* (p. 17, lines 272–274)

Although we attempted to generate reassortant viruses that combined mutations in PA with those in other genes (PB2 and NP) several times, we failed to rescue them. To confirm the association between mouse-adapted mutations in RNP complex and enhanced pathogenicity in mice, we are currently in the process of setting up a polymerase activity assay using minigenome plasmids containing the *Gaussia* luciferase reporter gene. An additional sentence was added to the results section to explain this.

*“Considering that complementary mutations to amino acid substitutions in PA occurred among other genes in RNP complex, we attempted to rescue the reassortant viruses that combined mutations in PA with those in other genes in the RNP complex, such as PB2+PA, PA+NP, and PB2+PA+NP; however, these strains could not be resucoed for futher evaluation.”* (p. 16, lines 259–263)

We agree with the reviewer's comment that it is necessary to reproduce the MA_SW characteristics closer to perfection through a combination of mutations derived from different genes as all mutant viruses presented lower virulence in mice than that of MA_SW. However, the mutant viruses showed outstanding mouse adaptation compared to the wild-type virus (RG_SW). In particular, the HA mutant showed faster growth rate than that of MA_SW in MDCK cells (Fig 4), confirming that the mutations are important determinants of infectivity. The PB2 mutant and NP mutant presented enhanced replication competence in mouse and MDCK cells compared to those of RG_SW (Figs 3 and 4). Mutations in PB2 and NP may be considered as factors related to replication competence. Although the MA_SW characteristics were not completely reproduced in this study, it can be confirmed that the factors identified in the study are important for infection and replication of seasonal H3N2 influenza in mice.

**Major point 5:** Since the virus of PA mutation alone was not rescuable, at least a combination of PA with other mutations could have been tried. Since the D34N of NP is frequently found in MA H3N2, combinations of the NP mutation with PA and PB2 mutations could have been tested. If all mutations were found in the same colony (there is no information about this in current manuscript!), please do the mutation combination experiments.

**Response:** As mentioned in the response to major point 4, we had tried to generate reassortant viruses that combined mutations in PA with those in other genes (PB2 and NP), but failed to rescue them. To confirm the association between mouse-adapted mutations in RNP complex and enhanced pathogenicity in mice, we are currently in the process of setting up a polymerase activity assay using minigenome plasmids containing the *Gaussia* luciferase reporter gene. An additional sentence was added to the results section to explain this.

*“Considering that complementary mutations to amino acid substitutions in PA occurred among other genes in RNP complex, we attempted to rescue the reassortant viruses that combined mutations in PA with those in other genes in the RNP complex, such as PB2+PA, PA+NP, and PB2+PA+NP; however, these strains could not be resucoed for futher evaluation.”* (p. 16, lines 259–263)

**Minor point 1:** Is the amino acid position numbering a CDS numbering (not clear especially for HA)?

**Response:** We agree with the reviewer and understand the potential for misunderstanding the numbers. Therefore, we have added a sentence explaining the numbering in the results section.

*“Amino acid positions are numbered based on translated amino acids of DNA sequences encoding SW293 proteins (EPI_ISL_166310).”* (p. 13, lines 228–229)

**Minor point 2:** Line 146, title of Table 1 is wrong. Reorganizing the mutations by passage stage (i.e., passage in DBA/1J or Balb/c) is recommended.

**Response:** Following the reviewer’s request, Table 5 presenting the mutations by passage stage in Balb/c or DBA/1J was added instead of reorganizing Table 1 (it was changed to Table 7 in the revised manuscript).

**Minor point 3:** Lines 152 – 157, please try to write more clearly.

**Response:** As suggested by the reviewer, the sentence was modified in the results section.

*“In the case of MA_SW, higher doses were not used as 10^3^ TCID_50_ showed sufficient lethality in mice.”* (p. 17, lines 273–274)

**Minor point 4:** Lines 168 – 169, description doesn’t agree with the data.

**Response:** Following the reviewer’s suggestion, the sentence was modified in the results section.

*“however, the mortality rate of mice infected with 10^5^ of the HA mutant was at 40% (Fig 2C).”* (p. 18, lines 287–288)

**Minor point 5:** Lines 239 – 241, where is the reference?

**Response:** The sentence was modified as the mouse adaptation of SW293 data were added in the revised manuscript.

*“We generated a mouse-adapted H3N2 virus [maSW293B8 (MA_SW)] derived from A/Switzerland/9715293/2013 by serial lung-cell-lung passages in Balb/c and DBA/1J mice, which resulted in higher mortality rates in mice (Table 4 and S1 Table).”* (p. 21, lines 372–374)

**Minor point 6:** Line 243 – 245, check ‘nine amino acid substitutions’ and ‘seroconversion’ if they are correct.

**Response:** There were 13 amino acid substitutions in five proteins of MA_SW, but we failed to rescue PA mutants encompassing four mutations. Therefore, the mutant viruses rescued in this study contain 9 amino acid substitutions. The word ‘seroconversion’ was deleted because it was mistyped. We agree with the reviewer’s concern for potential misleading of readers, and therefore, the sentence was modified in the revised manuscript.

“*We rescued four reassortant viruses containing nine amino acid substitutions detected in the PB2, HA, NP, and NA genes of MA_SW except for the PA gene and evaluated their virulence, replication competence, and histopathology in mice.*” (p. 22, lines 377–379)

**Minor point 7:** Lines 275 – 279, the authors appear to be mixed up about mouse and human.

**Response:** The sentences were replaced by the revised ones following the reviewer’s comment.

*“Although the NP mutant showed low pathogenicity in mice (Figs 1D and 2D), it presented high replication efficiency in the mouse organs (Fig 3E) and MDCK cells (Fig 4), and severe histopathological lesions in the lung (Fig 5). In previous studies, virulence of the reassortant virus containing NP D34N was enhanced by increasing the dose inoculated into mice [9, 40]. Therefore, the pathogenicity of the NP mutant could be enhanced by increasing the dose. Taken together, NP D34N may be considered as a determinant of IAV pathogenicity.”* (p. 23, lines 411–417)

**[Reviewer #2]**

**Comment 1:** It would be great if passage numbers of appearance of mutations are presented. The order of mutations of each gene, especially HA, will provide a valuable insight into tracking mutations during the host adaptation.

**Response:** We did not conduct sequence analysis every passage step. However, we add Table 5 to present the mutations of five Balb/c- or DBA/1J-passaged clones showing high pathogenicity in mice. Furthermore, the data related to mouse adaptation of seasonal H3N2 influenza (A/Switzerland/9715293/2013, SW293) was added in Tables 1-4.

**Comment 2:** Lines 275~279: The authors stated that NP mutant virus was safe and the mutation might be a strategy to attenuate the virus. However, Figure 3 and Figure 4 show that NP mutant virus has increased ability to replicate in several organs as compared to the parent virus. Also, given that the NP mutation was induced by adaptation process in mice, the mutation is likely to be associated with increased virulence. The statement should be rephrased.

**Response:** The sentences were modified based on the reviewer’s comment.

*“Although the NP mutant showed low pathogenicity in mice (Figs 1D and 2D), it presented high replication efficiency in the mouse organs (Fig 3E) and MDCK cells (Fig 4), and severe histopathological lesions in the lung (Fig 5). In previous studies, virulence of the reassortant virus containing NP D34N was enhanced by increasing the dose inoculated into mice [9, 40]. Therefore, the pathogenicity of the NP mutant could be enhanced by increasing the dose. Taken together, NP D34N may be considered as a determinant of IAV pathogenicity.”* (p. 23, lines 411–417)

**Comment 3:** The time-dependent growth kinetics of each mutant virus on a cell culture (such as MDCK cell) would provide a better interpretation on replication abilities of the viruses. Especially, it would be interesting to examine whether delayed replication of the PB2 mutant in vivo (Fig 3) can be reproduced in vitro cell culture.

**Response:** Following the reviewer’s suggestion, the growth curve of mutants, RG_SW, and MA_SW were confirmed in MDCK cells and are presented in new Fig 4. This experiment is described in the M&M, the results, the discussion sections.

*“To analyze the time-dependent growth profiles of reassortant H3N2 viruses, MDCK cells were infected at a multiplicity of infection (MOI) of 0.001 and were incubated at 37°C. The supernatants were harvested at 3, 6, 12, 24, 48, and 72 h. The infectious viral titers were determined by TCID_50_ in MDCK cells.”* (p. 9, lines 164–167)

*“Cell growth properties of reassortant H3N2 viruses were examined in MDCK cells at 3, 6, 12, 24, 48, and 72 h post-infection (hpi) (Fig 4). All reassortant viruses and MA_SW showed higher peak titer and faster growth rate than those of RG_SW. The HA mutant presented the highest peak titer at 24 hpi and the fastest growth rate among the reassortant H3N2 viruses. The initial growth rate of the NP mutant was moderate; however, it recorded the second-highest peak titer following the HA mutant. The viral titer of the PB2 mutant was similar to that of the NP mutant until 24 hpi, after which the titer was maintained. These results indicated that mouse-adapted mutations contribute to the increased replication ability in mammalian cells.”* (p. 19–20, lines 325–332)

*“In MDCK cells, the initial growth rate of PB2 mutant was moderate until 6 hpi; however the mutant showed a rapid growth rate by 24 hpi (Fig 4).”* (p. 23–24, lines 422–423)

**Comment 4:** Line: 152~154: What does this sentence mean "We found that the MA_SW titer was lower than that of other viruses (3.16×104 TCID50/mL)…? Does it mean that the virus did not replicate to high titer enough in cell culture? Please clarify the meaning of this sentence.

**Response:** Based on the reviewer’s comment, the sentence was modified in the results section.

*“In the case of MA_SW, higher doses were not used as 10^3^ TCID_50_ showed sufficient lethality in mice.”* (p. 17, lines 273–274)
